# Repetitive Transcranial Magnetic Stimulation Over the Left Posterior Middle Temporal Gyrus Reduces Wrist Velocity During Emblematic Hand Gesture Imitation

**DOI:** 10.1007/s10548-018-0684-1

**Published:** 2018-11-08

**Authors:** Arran T. Reader, Nicholas P. Holmes

**Affiliations:** 1 Department of Neuroscience, Karolinska Institutet, Stockholm, Sweden; 2 Centre for Integrative Neuroscience and Neurodynamics, School of Psychology and Clinical Language Sciences, University of Reading, Reading, UK; 3 School of Psychology, University of Nottingham, Nottingham, UK

**Keywords:** Action recognition, Apraxia, Dual-route, Kinematics, Semantic storage, Two-person

## Abstract

Results from neuropsychological studies, and neuroimaging and behavioural experiments with healthy individuals, suggest that the imitation of meaningful and meaningless actions may be reliant on different processing routes. The left posterior middle temporal gyrus (pMTG) is one area that might be important for the recognition and imitation of meaningful actions. We studied the role of the left pMTG in imitation using repetitive transcranial magnetic stimulation (rTMS) and two-person motion-tracking. Participants imitated meaningless and emblematic meaningful hand and finger gestures performed by a confederate actor whilst both individuals were motion-tracked. rTMS was applied during action observation (before imitation) over the left pMTG or a vertex control site. Since meaningless action imitation has been previously associated with a greater wrist velocity and longer correction period at the end of the movement, we hypothesised that stimulation over the left pMTG would increase wrist velocity and extend the correction period of meaningful actions (i.e., due to interference with action recognition). We also hypothesised that imitator accuracy (actor-imitator correspondence) would be reduced following stimulation over the left pMTG. Contrary to our hypothesis, we found that stimulation over the pMTG, but not the vertex, during action observation reduced wrist velocity when participants later imitated meaningful, but not meaningless, hand gestures. These results provide causal evidence for a role of the left pMTG in the imitation of meaningful gestures, and may also be in keeping with proposals that left posterior temporal regions play a role in the production of postural components of gesture.

**Electronic supplementary material:**

The online version of this article (10.1007/s10548-018-0684-1) contains supplementary material, which is available to authorized users.

## Introduction

The lateral posterior temporal lobe, closely bordering the occipital lobe, is associated with high level visual perception, including the recognition of biological motion, tools, and body parts (Lingnau and Downing 2015). The left temporal lobe, particularly the middle temporal gyrus, is often considered to play a role in semantic storage (Binder et al. 2009) and retrieval (Davey et al. 2015), and damage to the left posterior middle temporal gyrus (pMTG) can result in apraxia (Buxbaum et al. 2014) or deficits in action recognition (Kalénine et al. 2010; Tarhan et al. 2015).

Action recognition may be particularly important for the imitation of meaningful actions, since our ability to imitate actions we are familiar with may rely on their extraction from long-term memory (Press and Heyes 2008; Tessari and Rumiati 2004). With this in mind, the left pMTG could be particularly important for meaningful action imitation. Notably, the pMTG is frequently reported in neuroimaging studies of imitation (Caspers et al. 2010), and might also be involved in recognising or knowing the meaning of observed intransitive actions (Kubiak and Króliczak 2016; Möttönen et al. 2016; Villarreal et al. 2008) and distinguishing between observed social and non-social actions (Wurm et al. 2017). Conversely, the left parietal lobe may be more important for meaningless action imitation (Buxbaum and Randerath 2018; Buxbaum et al. 2014; Goldenberg 2009; Rumiati et al. 2009).

In a previous experiment (Reader et al. 2018a), in which we motion-tracked participants’ wrist movements during meaningless and emblematic meaningful gesture imitation, we observed that the imitation of meaningless gestures was associated with a longer correction period than the imitation of meaningful gestures. That is, the deceleration phase of their wrist movements was longer, as reflected by a relatively earlier time to peak velocity (TPV/MT) and time to peak deceleration (TPD/MT), despite a longer overall movement time (MT). Participants also increased the speed (peak velocity, PV) of their movements during meaningless actions, perhaps reflecting a strategy designed to ensure time for a longer correction period when imitating actions that were unfamiliar (under time constraints). These results suggested that meaningful and meaningless action imitation can be distinguished by coarse-grain (wrist) kinematics. This is in keeping with neuropsychological reports (Petreska et al. 2007; Rumiati et al. 2009; Tessari and Cubelli 2014), neuroimaging (Decety et al. 1997; Menz et al. 2009; Peigneux et al. 2004; Rumiati et al. 2005; Tanaka et al. 2001), and other behavioural studies (Carmo and Rumiati 2009; Press and Heyes 2008; Tessari and Rumiati 2004) of imitation, which suggest that meaningful and meaningless action imitation may be reliant on separate routes. The modulation of these correction time parameters is likely to be strongly reliant on accurate apprehension of the observed action prior to imitation—whether it is meaningful or meaningless.

We hypothesised that repetitive transcranial magnetic stimulation (rTMS) over the left pMTG during action observation would influence kinematic correction time markers during subsequent imitation. Specifically, we hypothesised that, following stimulation over the pMTG, participants would perform meaningful actions more like meaningless actions (i.e., with earlier TPV/MT, TPD/MT, and greater PV and MT). We also expected that by stimulating over this area, we would reduce participant performance more in meaningful, than in meaningless, action imitation. This would be reflected in reduced imitation performance accuracy—the correspondence or correlation between the actor’s and the imitator’s behaviour.

## Materials and Methods

### Participants

We recruited 12 right-handed participants from the University of Nottingham and the surrounding area (mean ± SE age = 24.0 ± 1.04 years, 1 male). The experimental procedures were approved by the local ethics committee (ref: SoPEC 904); participants gave written, informed consent; and the experiments were conducted in accordance with the Declaration of Helsinki (as of 2008).

### Materials and Stimuli

The position of the participant’s right arm and hand and a confederate’s left arm and hand were recorded continuously using a wired Polhemus Liberty (Polhemus Inc., Colchester, VT, USA) 240 Hz, 16 channel (8 per person) motion-tracking system with 6 degrees of freedom (x, y, z, azimuth, elevation, and roll). Trackers were attached to the shoulder (acromial end of clavicle), elbow (olecranon), wrist (pisiform), and the tips of the thumb and fingers. Tracking points were attached using adhesive medical tape or Velcro™.

TMS was applied using a Magstim Rapid^2^ (The MagStim Company, Cardiff, UK) with one of two 75 mm outer diameter figure-of-eight precision coils. For recording motor-evoked potentials to find the resting motor threshold (RMT) in the first dorsal interosseus (FDI), and as a safety measure during stimulation (i.e., to monitor for potential seizure-related activity, Rossi et al. 2009), muscle activity was recorded continuously over the right FDI and brachioradialis using an AD Instruments Powerlab 16/30 sampling at 2 kHz via a Dual Bioamp/stimulator and LabChart software, with 10-500 Hz bandpass filtering.

The experiment was controlled and data were acquired using custom software written in Labview (National Instruments). We used LabMan (custom in-house software) to document experiments, and the HandLabToolbox (available from https://github.com/TheHandLab), and MATLAB 2016b (Mathworks, Inc.) to pre-process data.

**Fig. 1:**
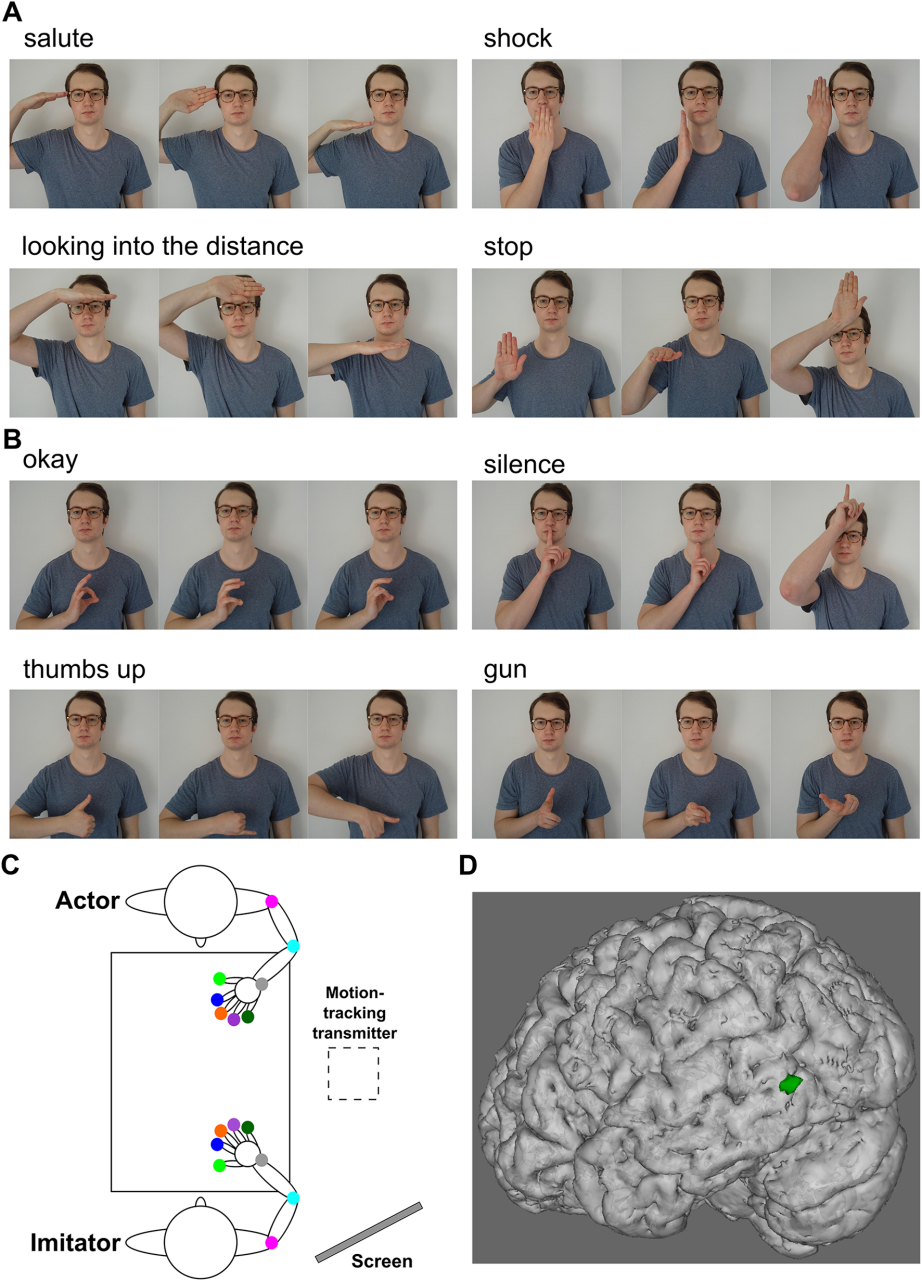
Stimuli, experimental setup, and rTMS site. **a** Hand gesture stimuli. For each meaningful gesture, two matched meaningless gestures were created. **b** Finger gesture stimuli. **c** Experimental set-up. Dots indicate the location of motion trackers. The tracking box was placed next to the table, and the confederate actor’s actions were cued through images displayed on a computer screen that was not observable to the participant imitator. **d** 95% confidence ellipsoid for the pMTG rTMS target site shown on a representative participant’s brain

A total of 24 gestures were used as stimuli. This included 4 meaningful hand gestures (“salute”, “shock”, “looking into the distance”, “stop”), 4 meaningful finger gestures (“okay”, “silence”, “thumbs up”, “gun”), and 16 matched meaningless gestures (Fig. 1a, b). In the case of finger gestures, the matching was done by changing the fingers used to create the gesture and/or the orientation or position of the hand. In the case of hand gestures, matching was done by either changing the orientation or position of the hand. In all analyses we used action effector (hand/finger) as a separate factor, since previous results (Reader and Holmes 2018) suggest that our hand gesture stimuli are generally imitated more accurately than the finger gestures.

During the imitation task, participants sat opposite a confederate actor at a rectangular plastic table, approximately 76 cm away from each other (Fig. 1c). A start point was located 20 cm away from each individual using Blu Tack®. In order to inform the confederate actor of the action they needed to perform, a computer screen was placed behind the imitator. This was unobservable by the participant imitator.

### Selection of TMS Sites

Visualisation of the participant’s brain was performed using T1-weighted MR images alongside the BrainSight stereotactic system (Rogue Research Inc., Montreal, QC, Canada). pMTG location was based on individual neuroanatomy rather than a statistical approach (Fig. 1d). The posterior limit of the MTG was designated by drawing an imaginary line from the pre-occipital notch vertically in the dorsal direction. The stimulation site was located halfway between the superior and middle temporal sulci, and approximately 10 mm from the posterior limit of the MTG. A control vertex stimulation site was found using normal measures (i.e., halfway between both the two pre-auricular points and the inion and nasion).

### TMS Parameters

Biphasic repetitive transcranial magnetic stimulation (rTMS) was applied over the pMTG and the vertex control for 2.67 s per trial at 3 Hz (i.e., nine pulses) and 110% of distance adjusted RMT (Stokes et al. 2007). For both stimulation sites, the coil was placed tangential to the skull. During pMTG stimulation, the coil was oriented between ~ 22.5° and ~ 67.5° in the sagittal plane (handle towards the back of the participant’s head), in order to ensure tangential placement dependent on the shape of each individual participant’s skull. During vertex stimulation, the handle was pointed towards the back of the participant’s head. Coil position was maintained throughout the experiment by securing it to a multi-joint arm attached to the ceiling.

Experimental stimulation intensity was limited to 80% of maximum stimulator output (MSO) to reduce coil overheating. RMT was obtained using the Rossini et al. (1994) method at the start of the first session, whilst participants were seated and relaxed. Mean ± SE RMT was 66 ± 2.9% MSO. To create the distance adjusted RMT, the distance from M1 and pMTG to the outside of the skull was measured using the BrainSight neuronavigation software. Vertex stimulation intensity was the same as pMTG. Mean ± SE experimental stimulation intensity was 66 ± 2.6% MSO.

### Design and Procedure

Participants took part in two sessions at least 24 h apart. In each session a single brain region was stimulated, with the order counterbalanced across participants. In every session, participants took part in both meaningful and meaningless action imitation tasks: one block of meaningful action imitation, and one block of meaningless action imitation. The block order was counterbalanced across sessions and participants. Meaningless and meaningful actions were presented in separate blocks, since there is some evidence to suggest that performing novel and known actions in a sequence recruits a single processing route, whilst presenting them separately recruits separate routes (Tessari and Cubelli 2014; Tessari and Rumiati 2004, but see; Press and Heyes 2008; Reader et al. 2018a). Hand and finger gestures were pseudorandomly interleaved within each separate block of action meaning. A trained male and female confederate were used, with each participant being assigned to one confederate for both of their testing sessions.

Both confederate actor and participant imitator began with their thumb and forefinger gripping their start points. In both meaningful and meaningless imitation tasks, action images were presented, in a random order, on a computer screen visible to the confederate but not the participant (Fig. 1c), which informed the confederate of which action to perform. A tone 1000 ms after the start of the image presentation signalled the actor to begin the action, which they performed and maintained until a second, lower pitched tone was played 2000 ms later. The actor then returned their hand to the start point. 1000 ms after the signal for the actor to return their hand, a higher pitched tone played to signal the imitator to copy the action, which they performed and maintained until a second, lower pitched tone was played 2000 ms later. The imitator then returned their hand to the start point. 64 trials were presented in this way, and the imitator was provided with a break at the halfway point. rTMS occurred during action observation, beginning 333 ms after the point at which the new image appeared on the screen. There was a 10 s gap between the end of one, and the start of the following train of stimulation, with trial timings matched to this criterion.

Following the completion of all TMS sessions, participants were presented with a questionnaire featuring the meaningful and meaningless images in a pseudorandom order. They were asked to state whether they thought each gesture had a meaning or not, and if it did to provide a brief explanation of the gesture. The purpose of this was to exclude participants if they were less than 60% consistent with our own categorisation of the actions, but no participants were excluded based on this criterion. Mean ± SE percentage agreement between participants and the experimenters’ categorisation was 86.5 ± 3.25% for meaningful actions and 82.8 ± 2.89% for meaningless actions. More specifically, the percentage agreement between participants and experimenters’ categorisation was 75.0 ± 5.33% for meaningful hand gestures, 97.9 ± 2.08% for meaningful finger gestures, 78.1 ± 3.81% for meaningless hand gestures, and 87.5 ± 3.44% for meaningless finger gestures.

### Data Analysis

Raw data are available from the Open Science Framework (10.17605/OSF.IO/EGBTR). An automated script was used for pre-processing and extraction of variables. The analysis routines processed the position data from each trial of each participant and rejected artefacts. Single timepoint spikes (> 3 SD from the within-trial mean), in each trial’s double-differentiated time-series were deemed electromagnetic artefacts and removed by interpolation across three adjacent samples either side.

The data were filtered with a bidirectional low-pass 4th order Butterworth filter (cutoff frequency 12 Hz). Trials in which either the actor or imitator moved for less than 400 ms, started before the starting tone, or failed to finish the action before the end of the trial, were excluded. Finally, all trials were visually inspected for remaining artefacts and excluded if any remained. Following the above exclusions, a total of 78.7% of trials were maintained for statistical analysis.

In keeping with Reader et al. (2018a), four imitator wrist kinematic variables were extracted: MT, PV, TPV/MT, and TPD/MT. The mean values of these variables across every trial for each condition were analysed using repeated-measures ANOVAs with three levels: stimulation site (pMTG, vertex), action meaning (meaningful, meaningless), and action effector (hand, finger). Bonferroni-correction was used for paired comparisons, when necessary, in the event of statistically significant interactions. Since some single trackers (other than the wrist) had remaining artefacts, we removed these trackers trial-wise in each instance. This resulted in 157 (0.81%) tracker-specific time-series removed from the analysis outlined below.

To test imitation accuracy, we compared the actor and the imitator 3D velocity (i.e., the change in 3D position) for each of the trackers over their primary movement (movement onset to gesture completion). To do this we ran a cross-correlation analysis between the original actor and imitator velocity curves for each trial and for each tracker, across lags of the difference between the actor and imitator timeseries length (i.e., if actor movement duration was 180 samples, and imitator movement duration was 240 samples, then cross-correlation was performed over lags of 1 sample steps from − 60 to + 60 samples). From this information we took the maximum r-value and the associated lag (i.e., the point at which the imitator’s velocity profile was best correlated with that of the actor) for each trial. To allow parametric analysis the resulting r-values were converted to Z-values using the Fisher transformation (Z = 0.5*ln(1 + r/1 − r), where ln is the natural logarithm). The means of the Z-values and lags (in milliseconds) for each condition were analysed using two three-way (stimulation site, meaning, effector) repeated measures ANOVAs.

To reduce our likelihood of reporting false positives in the multi-tracker Z-value and lag analyses, we divided our alpha value cutoff for assessing statistical significance by 8 (the number of trackers). Therefore in the ANOVAs of maximum Z-value and associated lag for each tracker, the alpha used to determine a significant result was reduced from .05 to .00625.

## Results

### Wrist Kinematics

**Fig. 2:**
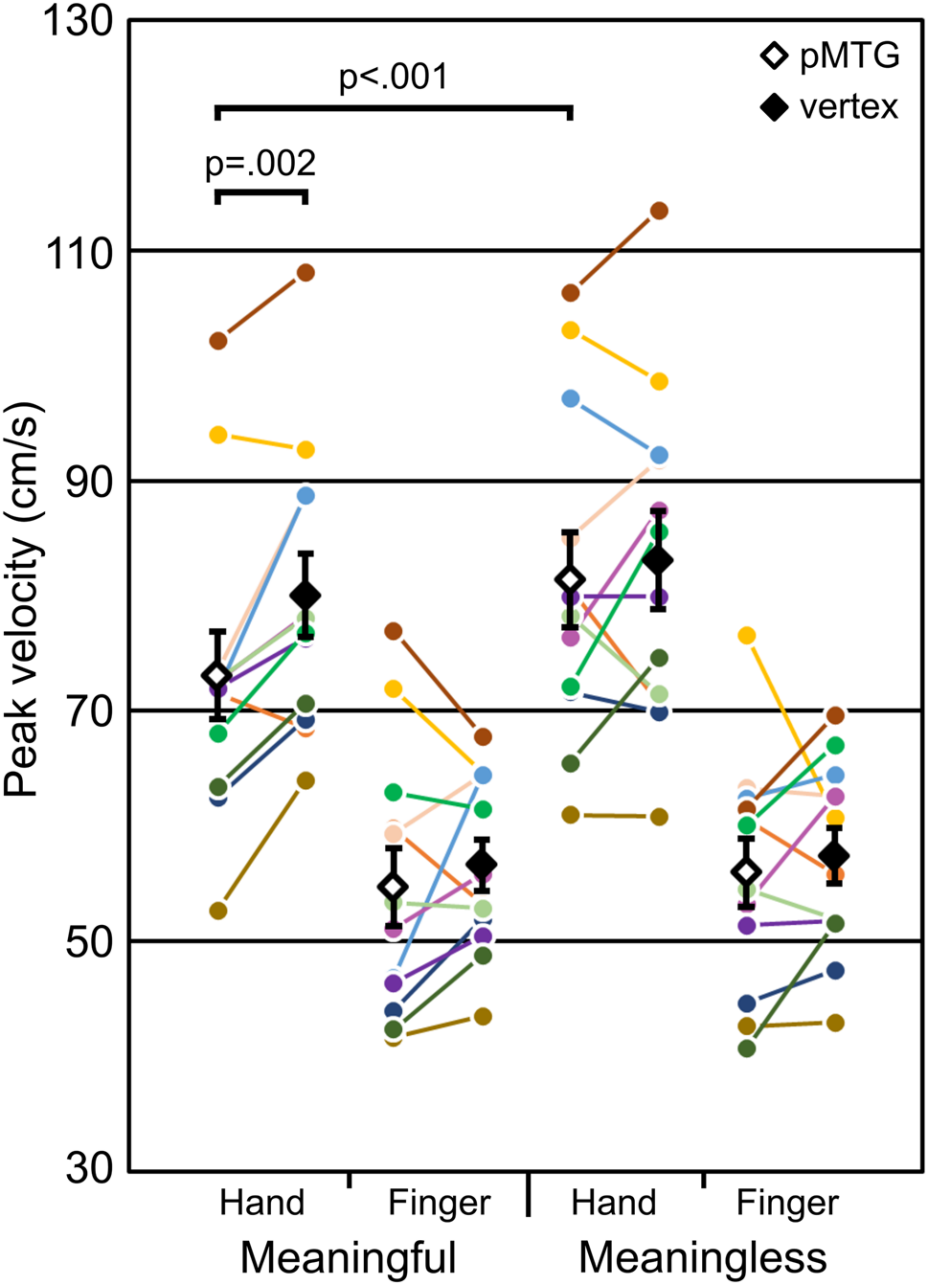
Paired comparisons for imitator wrist peak velocity. Diamonds indicate mean values; error bars indicate between-participant standard error; coloured circles indicate individual participant values for each condition

We observed a statistically significant site*meaning*effector interaction in wrist PV [F(1,11) = 8.36, p = .015, ƞ^2^ = .432] (Fig. 2). We examined this statistically significant interaction by comparing PV at the level of stimulation site and action meaning using eight two-tailed paired *t* tests. We used a Bonferroni-corrected alpha of .00625 in order to assess statistical significance for these paired comparisons.

In support of pMTG involvement in meaningful action imitation, but not in keeping with our original hypothesis, we found that wrist PV was significantly reduced for meaningful hand gestures following stimulation over the pMTG compared to over the vertex [t(11) = − 4.15, p = .002, g_rm_ = 0.497]. In addition, PV was significantly reduced for meaningful hand gestures compared to meaningless hand gestures following pMTG stimulation [t(11) = − 4.74, p < .001, g_rm_ = 0.555]. This pattern of effects was observed in 10 out of 12 of participants. Importantly, we did not observe a significant site*meaning*effector interaction for the actor’s wrist PV [F(1,11) = 2.54, p = .139, ƞ^2^ = .188], suggesting that the effects of rTMS could not necessarily be explained by biased actor behaviour. Furthermore, participant-wise confederate behaviour in the same direction of the statistically significant paired imitator effects was only observed in four cases.

There was no significant difference in wrist PV between pMTG and vertex stimulation for meaningful finger gestures [t(11) = − 0.884, p = .396, g_rm_ = 0.161], meaningless hand gestures [t(11) = − 0.756, p = .466, g_rm_ = 0.107], or meaningless finger gestures [t(11) = − 0.690, p = .505, g_rm_ = 0.141]. There was no significant difference in PV between meaningful and meaningless finger gestures following rTMS over the pMTG [t(11) = − 0.610, p = .554, g_rm_ = 0.103]. There was also no significant difference in PV between meaningful and meaningless finger gestures following rTMS over the vertex [t(11) = − 0.785, p = .449, g_rm_ = 0.0888], or in PV between meaningful and meaningless hand gestures following rTMS over the vertex [t(11) = − 2.34, p = .039, g_rm_ = 0.182].

Since the statistically significant effects of rTMS over pMTG on wrist velocity were only observed for hand gestures, we wanted to ensure that this was not just because the wrist tracker better characterised coarse-grain hand movements. We therefore decided post-hoc to also examine PV for the digits. In order to do this we subtracted the wrist position at each time point from the digit positions at each time point, in order to assess solely the digit movement element during the MT of each trial. We took the mean digit PV (i.e., across the five digits) for each condition and for each participant, and performed a repeated measures ANOVA as reported above. However, we found no statistically significant site*meaning*effector interaction, as had been observed for wrist PV (Supplemental Tables 1 and 2). We did, however, observe that the digits moved significantly slower in finger gestures [mean ± SE mean digit PV = 66.3 ± 3.17 cm/s] compared to hand gestures (72.6 ± 4.96 cm/s), F(1,11) = 5.94, p = .033, ƞ^2^ = .350. This was probably because finger gestures required more controlled positioning of the digits.

Beyond the effects related to stimulation site, we also observed that, compared to meaningful actions, meaningless actions had a significantly smaller wrist TPV/MT and TPD/MT, along with a significantly greater wrist PV and MT (Table 1). This replicates our previous findings (Reader et al. 2018a), which indicate that meaningless action imitation is associated with an increase in velocity in order to maintain a greater part of the movement in the correction phase, i.e., following peak deceleration.

**Table 1:** Mean values and main effects for wrist kinematic variables

Variable	Mean (± SE) value						Main effect								
	Site		Meaning		Effector		Site			Meaning			Effector		
	pMTG	Vertex	MF	ML	Hand	Finger	F(1,11)	p	ƞ^2^	F(1,11)	p	ƞ^2^	F(1,11)	p	ƞ^2^
PV (cm/s)	66.4 (3.32)	69.3 (3.00)	66.1 (3.05)	69.5 (3.15)	79.4 (3.80)	56.2 (2.51)	3.84	.076	.258	13.5	**.004**	.551	138	<** .001**	.926
TPV/MT (0–1)	.410 (.00654)	.408 (.00846)	.419 (.00756)	.398 (.00716)	.397 (.00826)	.421 (.00675)	0.231	.640	.021	46.8	<** .001**	.810	29.1	<** .001**	.726
TPD/MT (0–1)	.684 (.0102)	.689 (.00839)	.699 (.00977)	.675 (.00860)	.676 (.00793)	.697 (.0115)	0.428	.526	.037	9.02	**.012**	.451	3.56	.086	.245
MT (ms)	800 (18.5)	808 (21.7)	785 (19.4)	823 (20.5)	865 (22.7)	743 (18.5)	0.690	.424	.059	22.9	**.001**	.675	79.3	<** .001**	.878

Statistically significant p-values are in bold. Mean values for all conditions in Supplemental Table 3. Interactions in Supplemental Table 4

*PV* peak velocity, *TPV*/*MT* time to peak velocity/movement time, *TPD*/*MT* time to peak deceleration/movement time, *MT* movement time, *MF* meaningful, *ML* meaningless

Compared to finger gestures, hand gestures had a significantly greater wrist PV and MT, and significantly smaller TPV/MT (Table 1), suggesting, generally, that during hand gestures the wrist moved with a higher speed but for a longer duration. Statistically significant meaning*effector interactions (Supplemental Table 4) were also observed in PV [F(1,11) = 25.3, p < .001, ƞ^2^ = .697], TPV/MT [F(1,11) = 6.93, p = .023, ƞ^2^ = .386] and MT [F(1,11) = 27.8, p < .001, ƞ^2^ = .717]. We examined these statistically significant interactions using two-tailed paired *t* tests.

Wrist PV was significantly greater in meaningless hand (82.3 ± 4.04 cm/s) compared to meaningful hand (76.6 ± 3.63 cm/s) gestures [t(11) = 5.50, p < .001, g_rm_ = 0.367]. However, there was no significant difference between meaningless finger (56.7 ± 2.48 cm/s) and meaningful finger (55.7 ± 2.63 cm/s) gestures [t(11) = 0.996, p = .341, g_rm_ = 0.105]. Wrist TPV/MT was significantly smaller in meaningless hand (.381 ± .00754) compared to meaningful hand (.412 ± .00943) gestures [t(11) = − 7.07, p < .001, g_rm_ = 0.870]. There was no significant difference in TPV/MT between meaningless finger (.415 ± .00745) and meaningful finger (.426 ± .00703) gestures [t(11) = − 2.04, p = .066, g_rm_ = 0.407]. Finally, wrist MT was significantly greater in meaningless hand (897 ± 23.4 ms) compared to meaningful hand (833 ± 23.0 ms) gestures [t(11) = 6.48, p < .001, g_rm_ = 0.733]. However, there was no significant difference between meaningless finger (750 ± 19.0 ms) and meaningful finger (736 ± 19.0 ms) gestures [t(11) = 1.51, p = .161, g_rm_ = 0.188]. These results suggest that the significant main effects of action meaning in wrist PV, TPV/MT, and MT may have been driven by differences between meaningful and meaningless hand gestures.

### Actor-Imitator Correspondence

There were significant main effects (Supplemental Table 5), but not interactions (Supplemental Table 6) for maximum Z-value, representing the correlation between actor and imitator tracker velocity profiles. Hand gestures were significantly better correlated between actor and imitator than finger gestures in most trackers: shoulder, elbow, wrist, thumb, index, and little fingers (in all cases F(1,11) > 18, p ≤ .001, ƞ^2^ > .600). These effects, suggesting greater imitation accuracy for our hand gestures, replicate previous work (Reader and Holmes 2018). Meaningless gestures were significantly less correlated than meaningful gestures in the little finger [F(1,11) = 15.5, p = .002, ƞ^2^ = .585], but this statistically significant effect was not found in any other trackers. There were no significant main effects (Supplemental Table 7) or interactions (Supplemental Table 8) observed for lag at maximum Z-value.

## Discussion

In this experiment we examined the role of the left pMTG in meaningful and meaningless action imitation. We hypothesised that rTMS over the left pMTG during action observation would impair the recognition function associated with this region, and cause participants to perform meaningful actions more akin to meaningless actions (i.e., with a longer correction period, reflected in a proportionally earlier wrist PV and peak deceleration, and greater MT and PV). In addition, we expected that, following stimulation over the pMTG, participants would show reduced accuracy in meaningful, but not meaningless, action imitation, which would be reflected in reduced actor-imitator correspondence. We did not observe results in support of either of these hypotheses. Instead, we found that stimulation over the left pMTG reduced the wrist velocity with which participants imitated meaningful hand gestures.

### pMTG and Meaningful Hand Gestures

Our most notable finding was that stimulation over the left pMTG resulted in a significantly reduced wrist speed (PV) for the performance of meaningful hand gestures, but not meaningless hand gestures, compared to stimulation over the vertex control site. We expected PV to increase in this scenario, considering that in a previous experiment (Reader et al. 2018a) we observed that when participants imitate meaningless actions, their wrist moves with greater velocity, possibly to increase the correction time available prior to the final hand posture formation. We reasonably expected that interrupting the activity of an area involved in action recognition could lead participants to take an approach to imitation more like that observed for meaningless actions. However, whilst our main effects of action meaning were in keeping with previous findings, we did not observe any influence of rTMS over pMTG on these correction time markers.

In the light of a failure to support our original hypothesis, what might the effect of rTMS over the left pMTG represent? One possibility is that stimulation over the left pMTG during action observation did in fact interfere with action recognition, which in turn introduced a delay in the following imitative action performance. This possibility is supported by meta-analytic evidence suggesting that the pMTG is more frequently associated with action observation than action performance (Caspers et al. 2010; Hardwick et al. 2018; Grèzes and Decety 2001), or proposals that this region processes observed action kinematics (Hamilton 2008). However, the fact that this effect was observed solely for meaningful hand gestures, and not for finger gestures (even when directly assessing the movement of the digits, rather than the wrist), is harder to explain.

Previous discussions regarding the different requirements of hand and finger gesture imitation have generally occurred in the context of meaningless action imitation deficits in apraxia. These skills have frequently been assessed using established hand and finger gesture stimuli (Goldenberg 1996). In particular, these hand gestures rely on positioning the hand relative to other parts of the body, which may require breaking down the observed gesture into basic spatial relationships between body parts (Goldenberg 2001; Goldenberg and Karnath 2006). In contrast, the finger gestures require serial positioning of the digits with the hand position remaining consistent. Some have suggested that these tasks are strongly reliant on different areas of the brain (Goldenberg and Karnath 2006; Goldenberg and Randerath 2015; Goldenberg 2001, 2009), whilst others propose a shared network (Achilles et al. 2017). Our hand and finger gestures were not designed in keeping with these classical distinctions, and the focus on meaningless action in previous reports makes it hard for us to draw parallels with existing neuropsychological work (but see Achilles et al. 2016). However, given that our hand gesture stimuli clearly show greater wrist position variability compared to the finger gesture stimuli, it is feasible the effect of rTMS over the left pMTG on wrist velocity has something to do with the postural aspects of the imitation task (i.e., the hand position relative to other parts of the body).

One interesting possibility is that a residual effect of the stimulation performed during action observation may have reduced the efficiency with which participants could use stored information regarding familiar (meaningful) hand postures during action. This would be in keeping with some previous neuroimaging work (e.g., Astafiev et al. 2004; Dinstein et al. 2007; Gallivan et al. 2016; Johnson-Frey et al. 2005; Lingnau and Downing 2015; Króliczak and Frey 2009; Oosterhof et al. 2010), and also suggestions from neuropsychological reports that the left posterior temporal lobe is involved in transitive movements in an imitative scenario or to demand (Buxbaum et al. 2014; Tarhan et al. 2015; but see; Vingerhoets and Clauwaert 2015). Notably, some have suggested that the left posterior temporal lobe is important for ‘the production of postural aspects of tool-related actions’ (Buxbaum et al. 2014, p. 1981). Our results suggest that this capacity may extend to intransitive, emblematic action performance. Whilst the left posterior temporal lobe may also be involved in the kinematic aspects of gesture (Buxbaum et al. 2014), it is important to note that our observed change in PV following rTMS over the pMTG may not be due to direct interference with kinematic processing. Rather, the reduced movement speed may reflect reduced certainty regarding the final hand position when the retrieval of postural information is impaired following stimulation.

The absence of TMS-related effects in finger gesture performance could be because our dataset or analysis may have been more sensitive to detect differences in hand gestures than in finger gestures. For example, if our effects of rTMS are due to interference with postural production, the postural components of finger gestures (i.e., their relative positions during gesture formation or at gesture completion) may not have been adequately captured by our stimuli or analysis. Alternatively, it could be that the organisation of semantic information regarding emblematic hand and finger gestures is segregated in such a way that our stimulation only interfered with hand gestures. The categorical organisation of information in occipitotemporal regions (Bracci et al. 2010, 2015, 2017; Downing et al. 2007; Lingnau and Downing 2015; Wurm and Lingnau 2015) might support this, though we are not convinced that our neuro-navigation approach is specific enough for such an effect.

### Imitation Accuracy

As in a previous experiment (Reader and Holmes 2018), our actor-imitator correspondence analysis revealed that hand gestures were more accurately imitated (i.e., with greater correlation between actor and imitator velocity profiles) compared to finger gestures. The absence of TMS site-related effects in this actor-imitator correspondence suggests that rTMS may not be sufficiently disruptive to influence imitation accuracy, compared to the deficits that can be observed following large scale lesion damage. This is also in keeping with what we have observed with similar experimental methods (Reader and Holmes 2018; Reader et al. 2018b), which suggest that rTMS over areas associated with imitation results in relatively subtle changes in kinematics, rather than changes in imitation accuracy as assessed by the correlation between actor and imitator velocity profiles.

### Effects of Action Meaning and Effector on Wrist Kinematics

Finally, wrist kinematics showed similar effects of action meaning as previously reported. Specifically, meaningless actions had a significantly smaller TPV/MT and TPD/MT, along with a significantly greater PV and MT compared to meaningful actions (i.e., the correction time markers reported in Reader et al. 2018a).

Furthermore, we observed that meaningless hand gestures had a significantly greater PV than meaningful hand gestures, whilst meaningful and meaningless finger gestures were not significantly different. We also found that TPV/MT was significantly smaller in meaningless compared to meaningful hand gestures, whilst MT was significantly longer in meaningless hand compared to meaningful hand gestures. As with PV, similar statistically significant effects were not observed for finger gestures. This seems to suggest that correction time strategies in meaningless actions are better captured in our hand gesture stimuli, perhaps because of the greater distance that the hand must move (mean ± SE distance moved for wrist tracker was 26.9 ± 1.10 cm for hand gestures, 18.9 ± 0.725 cm for finger gestures). It might be useful in future to assess whether similar kinematic markers can be observed in the digits during finger gesture formation.

## Conclusion

Our results provide causal evidence in healthy individuals for a role of the left pMTG in the imitation of meaningful (emblematic) hand gestures, which may support a role for the left posterior temporal lobe in producing known postural configurations (i.e., placing the hand relative to other parts of the body). However, more work is needed to better clarify this, and also better examine interactions between action meaning and action effector during imitation.

## Electronic supplementary material

Below is the link to the electronic supplementary material.

Supplementary material 1 (DOC 124 KB)
