# Supplementary material for: Repetitive Transcranial Magnetic Stimulation Over the Left Posterior Middle Temporal Gyrus Reduces Wrist Velocity During Emblematic Hand Gesture Imitation
Source: Brain Topogr. 2018 Nov 8;32(2):332–41. doi: 10.1007/s10548-018-0684-1 (PMC6373290; doi:10.1007/s10548-018-0684-1)
Supplement: Supplementary file 1 — Supplementary material 1 (DOC 124 KB) [file 10548_2018_684_MOESM1_ESM.doc]

Supplemental material: Repetitive transcranial magnetic stimulation over the left posterior middle temporal gyrus reduces wrist velocity during emblematic hand gesture imitation

Arran T. Reader & Nicholas P. Holmes

**Supplemental Table 1: Mean values and main effects for mean digit peak velocity**

**Significant p-values are in bold.**

| **Mean(±SE) peak velocity in cm/s** | | | | | | **Main effect** | | | | | | | | |
| --- | --- | --- | --- | --- | --- | --- | --- | --- | --- | --- | --- | --- | --- | --- |
| **Site** | | **Meaning** | | **Effector** | | **Site** | | | **Meaning** | | | **Effector** | | |
| **pMTG** | **Vertex** | **MF** | **ML** | **Hand** | **Finger** | **F (1, 11)** | **p** | **ƞ^2^** | **F (1, 11)** | **p** | **ƞ^2^** | **F (1, 11)** | **p** | **ƞ^2^** |
| 69.1  (3.87) | 69.8  (4.36) | 70.4  (3.89) | 68.5  (4.09) | 72.6  (4.96) | 66.3  (3.17) | 0.101 | .756 | .009 | 3.23 | .100 | .227 | 5.94 | **.033** | .350 |

**Supplemental Table 2: Interactions for mean digit peak velocity**

| **Site*meaning** | | | **Site*effector** | | | **Meaning*effector** | | | **Site*meaning*effector** | | |
| --- | --- | --- | --- | --- | --- | --- | --- | --- | --- | --- | --- |
| **F (1, 11)** | **p** | **ƞ^2^** | **F (1, 11)** | **p** | **ƞ^2^** | **F (1, 11)** | **p** | **ƞ^2^** | **F (1, 11)** | **p** | **ƞ^2^** |
| 0.209 | .657 | .019 | 1.30 | .279 | .105 | 1.40 | .263 | .113 | 0.680 | .427 | .058 |

**Supplemental Table 3: Mean values for wrist kinematic variables in each condition**

| **Variable** | **Mean(±SE)** | | | | | | | |
| --- | --- | --- | --- | --- | --- | --- | --- | --- |
|  | **pMTG**  **Meaningful**  **Hand** | **pMTG**  **Meaningful**  **Finger** | **pMTG**  **Meaningless**  **Hand** | **pMTG**  **Meaningless**  **Finger** | **Vertex**  **Meaningful**  **Hand** | **Vertex**  **Meaningful**  **Finger** | **Vertex**  **Meaningless**  **Hand** | **Vertex**  **Meaningless**  **Finger** |
| **PV**  **(cm/s)** | 73.1  (3.83) | 54.7  (3.36) | 81.4  (4.12) | 56.0  (2.97) | 80.0  (3.62) | 56.6  (2.22) | 83.1  (4.25) | 57.4  (2.40) |
| **TPV/MT**  **(0 – 1)** | 0.415  (0.00925) | 0.424  (0.00766) | 0.383  (0.00739) | 0.417  (0.00783) | 0.409  (0.0102) | 0.428  (0.00788) | 0.380  (0.00889) | 0.413  (0.0105) |
| **TPD/MT**  **(0 – 1)** | 0.693  (0.0130) | 0.702  (0.0173) | 0.653  (0.0141) | 0.688  (0.0175) | 0.684 (0.00923) | 0.716  (0.0113) | 0.677  (0.0101) | 0.681  (0.0130) |
| **MT**  **(ms)** | 835  (24.3) | 737 (16.9) | 889  (24.7) | 740 (18.1) | 832  (23.7) | 736  (23.5) | 905  (23.5) | 760  (22.1) |

**Supplemental Table 4: Interactions for wrist kinematic variables**

**Significant p-values are in bold. PV: peak velocity, TPV/MT: time to peak velocity/movement time, TPD/MT: time to peak deceleration/movement time, MT: movement time**

| **Variable** | **Site*meaning** | | | **Site*effector** | | | **Meaning*effector** | | | **Site*meaning*effector** | | |
| --- | --- | --- | --- | --- | --- | --- | --- | --- | --- | --- | --- | --- |
|  | **F (1, 11)** | **p** | **ƞ^2^** | **F (1, 11)** | **p** | **ƞ^2^** | **F (1, 11)** | **p** | **ƞ^2^** | **F (1, 11)** | **p** | **ƞ^2^** |
| **PV**  **(cm/s)** | 1.65 | .225 | .130 | 3.85 | .076 | .259 | 25.3 | **<.001** | .697 | 8.36 | **.015** | .432 |
| **TPV/MT**  **(0 – 1)** | 0.122 | .733 | .011 | 0.666 | .432 | .057 | 6.93 | **.023** | .386 | 0.676 | .429 | .058 |
| **TPD/MT**  **(0 – 1)** | 0.154 | .702 | .014 | 0.061 | .810 | .005 | 0.006 | .940 | .001 | 4.66 | .054 | .298 |
| **MT**  **(ms)** | 2.52 | .141 | .186 | 0.092 | .767 | .008 | 27.8 | **<.001** | .717 | 0.019 | .892 | .002 |

**Supplemental Table 5: Mean values and main effects for maximum Z-value**

**Significant p-values (<.00625) are in bold; SH: shoulder, EL: elbow, WR: wrist, TH: thumb, IN: index finger, MI: middle finger, RI: ring finger, LI: little finger.**

| **Tracker** | **Mean(±SE) Z-value** | | | | | | **Main effect** | | | | | | | | |
| --- | --- | --- | --- | --- | --- | --- | --- | --- | --- | --- | --- | --- | --- | --- | --- |
|  | **Site** | | **Meaning** | | **Effector** | | **Site** | | | **Meaning** | | | **Effector** | | |
|  | **pMTG** | **Vertex** | **MF** | **ML** | **Hand** | **Finger** | **F (1, 11)** | **p** | **ƞ^2^** | **F (1, 11)** | **p** | **ƞ^2^** | **F (1, 11)** | **p** | **ƞ^2^** |
| **SH** | 0.877  (0.0464) | 0.848  (0.0513) | 0.817  (0.0448) | 0.909  (0.0565) | 1.10  (0.0465) | 0.626  (0.0535) | 1.12 | .313 | .092 | 5.34 | .041 | .327 | 181 | **<.001** | .943 |
| **EL** | 1.62  (0.0825) | 1.62  (0.0800) | 1.62  (0.0805) | 1.62  (0.0760) | 1.83  (0.0702) | 1.40  (0.0878) | <.001 | .986 | <.001 | 0.008 | .931 | .001 | 48.5 | **<.001** | .815 |
| **WR** | 2.17  (0.0723) | 2.19  (0.0727) | 2.20  (0.0759) | 2.16  (0.0678) | 2.27  (0.0698) | 2.09  (0.0765) | 0.740 | .408 | .063 | 3.40 | .092 | .236 | 28.1 | **<.001** | .719 |
| **TH** | 1.81  (0.0543) | 1.88  (0.0516) | 1.86  (0.0604) | 1.83  (0.0415) | 1.95  (0.0541) | 1.74  (0.0548) | 5.07 | .046 | .316 | 2.08 | .177 | .159 | 22.9 | **.001** | .676 |
| **IN** | 1.76  (0.0562) | 1.82  (0.0413) | 1.80  (0.0531) | 1.78  (0.0402) | 1.92  (0.0601) | 1.66  (0.0361) | 1.43 | .257 | .115 | 0.189 | .673 | .017 | 36.6 | **<.001** | .769 |
| **MI** | 1.81  (0.0597) | 1.88  (0.0502) | 1.88  (0.0627) | 1.82  (0.0453) | 1.91  (0.0571) | 1.79  (0.0536) | 3.80 | .077 | .257 | 2.94 | .114 | .211 | 10.2 | .009 | .480 |
| **RI** | 1.91  (0.0611) | 1.96  (0.0584) | 1.96  (0.0641) | 1.90  (0.0516) | 1.98  (0.0644) | 1.88  (0.0553) | 1.69 | .221 | .133 | 6.73 | .025 | .380 | 7.55 | .019 | .407 |
| **LI** | 1.94  (0.0600) | 1.97  (0.0516) | 2.00  (0.0584) | 1.90  (0.0506) | 2.04  (0.0655) | 1.86  (0.0470) | 0.770 | .399 | .065 | 15.5 | **.002** | .585 | 18.7 | **.001** | .630 |

**Supplemental Table 6: Interactions for maximum Z-value**

SH: shoulder, EL: elbow, WR: wrist, TH: thumb, IN: index finger, MI: middle finger, RI: ring finger, LI: little finger.

| **Tracker** | **Site*meaning** | | | **Site*effector** | | | **Meaning*effector** | | | **Site*meaning*effector** | | |
| --- | --- | --- | --- | --- | --- | --- | --- | --- | --- | --- | --- | --- |
|  | **F (1, 11)** | **p** | **ƞ^2^** | **F (1, 11)** | **p** | **ƞ^2^** | **F (1, 11)** | **p** | **ƞ^2^** | **F (1, 11)** | **p** | **ƞ^2^** |
| **SH** | 0.325 | .580 | .029 | 0.011 | .919 | .001 | 0.024 | .879 | .002 | 1.03 | .332 | .086 |
| **EL** | 0.324 | .580 | .029 | 0.004 | .950 | <.001 | 0.160 | .697 | .014 | 1.21 | .296 | .099 |
| **WR** | 1.07 | .323 | .089 | 1.21 | .295 | .099 | 5.16 | .044 | .319 | 0.393 | .543 | .035 |
| **TH** | 0.174 | .685 | .016 | 0.153 | .703 | .014 | 0.040 | .845 | .004 | 0.016 | .900 | .001 |
| **IN** | 2.09 | .176 | .160 | 0.990 | .341 | .083 | 0.399 | .541 | .035 | 0.008 | .932 | .001 |
| **MI** | 1.17 | .304 | .096 | .215 | .652 | .019 | 6.00 | .032 | .353 | 0.936 | .354 | .078 |
| **RI** | 1.11 | .315 | .092 | 0.059 | .812 | .005 | 0.023 | .883 | .002 | 0.021 | .887 | .021 |
| **LI** | 2.96 | .114 | .212 | 0.001 | .979 | <.001 | 10.8 | .007 | .496 | 0.334 | .575 | .029 |

**Supplemental Table 7: Mean values and main effects for lag at maximum Z-value**

SH: shoulder, EL: elbow, WR: wrist, TH: thumb, IN: index finger, MI: middle finger, RI: ring finger, LI: little finger.

| **Tracker** | **Mean(±SE) lag in ms** | | | | | | **Main effect** | | | | | | | | |
| --- | --- | --- | --- | --- | --- | --- | --- | --- | --- | --- | --- | --- | --- | --- | --- |
|  | **Site** | | **Meaning** | | **Effector** | | **Site** | | | **Meaning** | | | **Effector** | | |
|  | **pMTG** | **Vertex** | **MF** | **ML** | **Hand** | **Finger** | **F (1, 11)** | **p** | **ƞ^2^** | **F (1, 11)** | **p** | **ƞ^2^** | **F (1, 11)** | **p** | **ƞ^2^** |
| **SH** | 7.08  (1.91) | 10.8  (3.14) | 8.10  (1.87) | 9.79  (2.84) | 15.5  (4.41) | 2.34  (2.81) | 0.887 | .366 | .075 | 0.243 | .632 | .022 | 6.28 | .029 | .363 |
| **EL** | 19.1  (5.16) | 13.3  (7.01) | 17.5  (4.47) | 15.0  (7.42) | 15.5  (5.49) | 16.9  (6.78) | 1.49 | .248 | .119 | 0.292 | .599 | .026 | 0.082 | .780 | .007 |
| **WR** | 33.2  (7.65) | 26.8  (11.2) | 34.3  (8.85) | 25.7  (10.4) | 36.9  (11.4) | 23.1  (8.95) | 1.08 | .321 | .089 | 1.87 | .199 | .145 | 2.15 | .170 | .164 |
| **TH** | 19.7  (7.55) | 19.0  (11.1) | 22.5  (9.21) | 16.2  (9.41) | 25.2  (10.8) | 13.5  (8.25) | 0.012 | .915 | .001 | 1.47 | .250 | .118 | 2.69 | .129 | .197 |
| **IN** | 18.9  (7.99) | 18.1  (10.6) | 22.3  (10.4) | 14.6  (8.58) | 20.8  (11.1) | 16.2  (8.78) | 0.022 | .886 | .002 | 1.41 | .259 | .114 | 0.257 | .622 | .023 |
| **MI** | 20.1  (7.42) | 20.8  (10.9) | 23.4  (9.61) | 17.5  (9.50) | 23.8  (11.5) | 17.1  (8.39) | 0.021 | .886 | .002 | 0.788 | .394 | .067 | 0.512 | .489 | .044 |
| **RI** | 17.6  (8.19) | 18.4  (12.1) | 20.9  (10.0) | 15.2  (10.6) | 22.3  (11.6) | 13.8  (9.66) | 0.015 | .906 | .001 | 0.815 | .386 | .069 | 1.02 | .334 | .085 |
| **LI** | 21.8  (8.50) | 22.1  (12.8) | 24.8  (11.5) | 19.1  (10.3) | 25.0  (12.0) | 18.9  (10.4) | 0.002 | .969 | <.001 | 0.745 | .406 | .063 | 0.509 | .490 | .044 |

**Supplemental Table 8: Interactions for lag at maximum Z-value**

SH: shoulder, EL: elbow, WR: wrist, TH: thumb, IN: index finger, MI: middle finger, RI: ring finger, LI: little finger.

| **Tracker** | **Site*meaning** | | | **Site*effector** | | | **Meaning*effector** | | | **Site*meaning*effector** | | |
| --- | --- | --- | --- | --- | --- | --- | --- | --- | --- | --- | --- | --- |
|  | **F (1, 11)** | **p** | **ƞ^2^** | **F (1, 11)** | **p** | **ƞ^2^** | **F (1, 11)** | **p** | **ƞ^2^** | **F (1, 11)** | **p** | **ƞ^2^** |
| **SH** | 0.104 | .753 | .009 | 1.11 | .315 | .091 | 0.274 | .611 | .024 | 0.196 | .667 | .017 |
| **EL** | 2.85 | .119 | .206 | 0.001 | .979 | <.001 | 2.31 | .157 | .174 | 0.316 | .585 | .028 |
| **WR** | 0.999 | .339 | .083 | 0.296 | .598 | .026 | 3.23 | .100 | .227 | 1.96 | .189 | .151 |
| **TH** | 0.619 | .448 | .053 | 0.014 | .909 | .001 | 1.36 | .268 | .110 | 0.468 | .508 | .041 |
| **IN** | 0.417 | .532 | .037 | 0.133 | .722 | .012 | 1.39 | 263 | .112 | 0.137 | .718 | .012 |
| **MI** | 6.13 | .031 | .358 | 0.028 | .870 | .003 | 1.07 | .322 | .089 | 0.399 | .541 | .035 |
| **RI** | 2.60 | .135 | .191 | 0.243 | .631 | .022 | 2.70 | .128 | .197 | 1.07 | .324 | .088 |
| **LI** | 2.12 | .173 | .162 | 0.002 | .964 | <.001 | 0.797 | .391 | .068 | 0.220 | .648 | .020 |
